# Following the Fate of Dye-Containing Liposomes In Vitro

**DOI:** 10.3390/ijms21144847

**Published:** 2020-07-09

**Authors:** Jennifer Cauzzo, Mona Nystad, Ann Mari Holsæter, Purusotam Basnet, Nataša Škalko-Basnet

**Affiliations:** 1 Drug Transport and Delivery Research Group, Department of Pharmacy, Faculty of Health Sciences, University of Tromsø The Arctic University of Norway, N-9037 Tromsø, Norway; jennifer.cauzzo@uit.no (J.C.); ann-mari.holsater@uit.no (A.M.H.); 2 Women’s Health and Perinatology Research Group, Department of Clinical Medicine, Faculty of Health Sciences, University of Tromsø The Arctic University of Norway, N-9037 Tromsø, Norway; mona.nystad@uit.no (M.N.); purusotam.basnet@uit.no (P.B.); 3 Department of Medical Genetics, University Hospital of North Norway, N-9038 Tromsø, Norway; 4 Department of Obstetrics and Gynecology, University Hospital of North Norway, N-9038 Tromsø, Norway

**Keywords:** liposomes, nanomedicine, cellular uptake, fluorescent dye, stability

## Abstract

The rather limited success of translation from basic research to clinical application has been highlighted as a major issue in the nanomedicine field. To identify the factors influencing the applicability of nanosystems as drug carriers and potential nanomedicine, we focused on following their fate through fluorescence-based assays, namely flow cytometry and imaging. These methods are often used to follow the nanocarrier internalization and targeting; however, the validity of the obtained results strictly depends on how much the nanosystem’s fate can be inferred from the fate of fluorescent dyes. To evaluate the parameters that affect the physicochemical and biological stability of the labeled nanosystems, we studied the versatility of two lipid dyes, TopFluor^®^-PC and Cy5-DSPE, in conventional liposomes utilizing well-defined in vitro assays. Our results suggest that the dye can affect the major characteristics of the system, such as vesicle size and zeta-potential. However, a nanocarrier can also affect the dye properties. Medium, temperature, time, fluorophore localization and its concentration, as well as their interplay, affect the outcome of tracing experiments. Therefore, an in-depth characterization of the labeled nanosystem should be fundamental to understand the conditions that validate the results within the screening process in optimization of nanocarrier.

## 1. Introduction

Nanomedicine has been proposed as the superior approach within advanced drug therapy, able to respond to the ever growing demands of various diseases. However, the past decades did not fully confirm its translational significance [1,2]. The reasons for this limited success rate are numerous and often interconnected, however, the heterogeneous physicochemical characteristics of nanomedicine formulations have a large influence on their biological performance [3]. In general, cellular uptake of drug-containing nanocarriers, including liposomes, will determine both the efficacy of drug delivery and toxic effects of carrier-associated drugs or active molecules. Liposomes made of natural phospholipids offer reassuring safety profiles [4] and become one of the most studied nanomedicines. Their versatility offers opportunities to tailor their features to enhance specific interactions with the target site. Optimizing liposomal features involves predicting/controlling their fate in vivo. The first step in optimization should, therefore, focus on determining the cellular uptake in rather simple in vitro conditions. There are various methods to follow and quantify the cellular uptake; probably, the most commonly applied methods are flow cytometry and imaging [5]. The fate of nanocarriers is often followed by tracing one or more fluorescent dyes associated with carriers. Based on the results, the formulations will be modified/tailored to achieve the desired cellular uptake. However, it is important to consider that the findings are based on two assumptions: i) the dye does not alter the interactions of nanocarriers with cells/tissues; ii) the dye encapsulated/incorporated in the nanocarrier is stable and remains associated with the carrier throughout the process. Unfortunately, these assumptions are not always correct [6]. Rodriguez-Lorenzo et al. [7] first described the possibility of the dye affecting the properties of the carrier, regarding gold nanoparticles. The change in surface charge due to the covalent link of a fluorescent dye had great effect on the cellular internalization. Considering liposomes as nanocarriers, liposomes are most often labeled by the fluorescent dye lipids incorporated within liposomal bilayers. Recently, Münter et al. [8] validated the assumption that dye remains associated with the liposomes throughout the experiment and found that the choice of labeled lipid can influence the conclusions on uptake of liposomes, as well as their localization within the cell. Many of the commonly used lipid dyes dissociate prior or upon exposure to biological environment. Moreover, the dye dissociations are not detected in the commonly applied in vitro assays used to determine dye leakage. For both (i) dye-induced modification of cellular fate and (ii) dye leakage, the dye loses its primary function of tracking the system behavior; as a result, the fate of the dye is followed rather than the fate of the dye-labeled nanocarrier. 

Rather than searching for an ever superior nanomedicine formulation, we propose to go back to basics and start rechecking the widely accepted dogmas. In an attempt to do so, we have focused on following the fate of liposomes as a model nanosized system in in vitro cell culture settings. Therefore, as the first step, we assessed the system’s stability in respect to both the effect that the dye might have on the physicochemical properties of the carrier, as well as the effect that the formulation might have on the dye stability. Finally, we investigated the biological properties of the new labeled nanocarrier.

Our hypothesis was that the dye-labeled nanocarrier can be considered as a new system, which needs to be compared to the dye-free delivery system and the free dye. To confirm our hypothesis, we chose to label our liposomes using fluorophores that resemble the chemical structure of the phospholipids building liposomes, and investigated their interference on the intrinsic behavior of the bilayer. Hence, we selected two commonly used fluorescent lipids [9], namely 1-palmitoyl-2-(dipyrrometheneboron difluoride)undecanoyl-sn-glycero-3-phosphocholine (TopFluor^®^-PC, T) and 1,2-distearoyl-sn-glycero-3-phosphoethanolamine-N-(Cyanine 5) (Cy5-DSPE, C) [6,8] as labels for our nanocarrier. Both dyes in four different ratios were individually incorporated in liposomes, and their stability, as well as impact on in vitro liposomal cellular uptake, were evaluated and compared.

## 2. Results

### 2.1. Liposome Characterization and Stability

**Figure 1:**
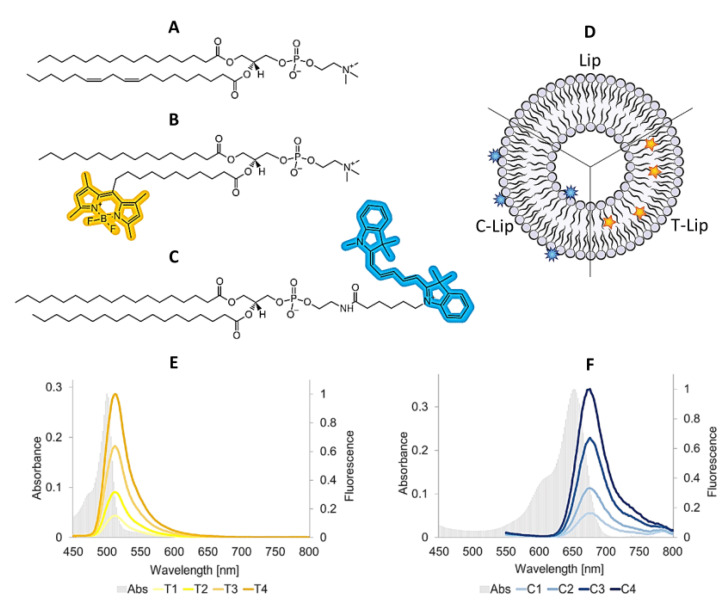
Molecular formulae of the lipid ingredients, postulated dye localization in the liposomal bilayer and fluorescence spectra of all dye-containing liposomal formulations (Day 0). The molecular formula of the most representative lipid in soy phosphatidylcholine (SPC) is provided (panel **A**) for comparison with the structures of the labeled phospholipids C and T (panel **B** and **C**, respectively). ACD/ChemSketch (Freeware) 2019 2.1 was used to draw the molecules and highlight the fluorescent moieties. All lipids ingredients are insoluble in water and highly soluble in methanol (respectively, >10000 and <1 mass parts of solvent required to dissolve 1 mass part of solute; according to Pharmacopeia’s definition). The liposome model (panel **D**) shows the expected localization of the labeled phospholipids according to their chemical structure and previous studies [10,11]. Panel **E** and **F** show the spectra for T-Lip and C-Lip formulations. The gray background spectrum represents the absorbance (primary y-axis), the solid colored lines refer to the fluorescent emission for all labeled formulations after normalization on the maximum values for T4 and C4 (secondary y-axis). Abbreviations: Lip refers to empty liposomes; T-Lip and C-Lip to the labeled formulations.

Composition, vesicle size, ζ-potential and fluorescence spectra for all freshly prepared formulations (Day 0) are summarized in Figure 1 and Table 1. The liposomes comprising the lipid dye (T, TopFluor^®^-PC) are presented as T1, T2, T3 and T4 (Lip-T), with increasing concentration of dye (T1 the lowest and T4 the highest ratio). Similarly, the liposomes comprising the surface lipid dye (C, Cy5-DSPE) are referred to as C1, C2, C3 and C4 (Lip-C), respectively. The T dye (MW= 909.97) is a fatty acid-labeled phospholipid that is expected to accommodate its fluorescent moiety within the bilayer, without significantly altering its structure [10,11]. The C dye (MW= 1266.20) is a headgroup-labeled phospholipid that is expected to expose the fluorescent moiety on the surface (Figure 1).

All labeled liposomes were in the size range below 200 nm, to assure that vesicles could be administered intravenously [12]. Since NICOMP distributions permit the presentation of bimodal size distributions (vesicle populations), rather than conventional Gaussian distributions, we set up the internal quality parameters to be i) polydispersity index (PdI) below 0.25 and ii) vesicle intensity above 80% for the most representative size peak. Both parameters, therefore, indicate the clear dominance of one vesicle population over the other. The Gaussian ζ-potential distribution estimated the surface charge to be mostly neutral for all formulations, confirming that the presence of either dye was not significantly interfering with the surface property of the liposomes. Furthermore, all liposomal suspensions were visually inspected before any analysis to assure that no precipitates were detected. The low values of polydispersity index indicated that all labeled liposomes were rather homogenous in size, with the majority of the vesicles of very similar size. Moreover, the incorporation of dye within the liposome did not affect the vesicle size (Table 1).

**Figure 2:**
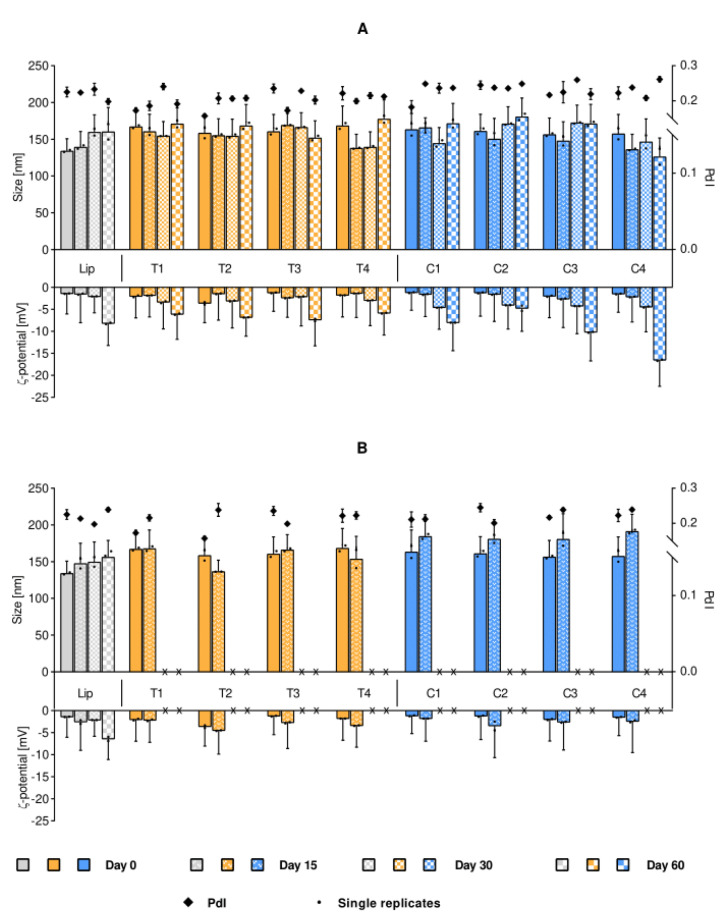
Stability of liposomal suspensions upon storage at 4 °C (panel **A**) and 25 °C (panel **B**). The stability is expressed as changes in original liposomal size and ζ-potential. Each bar cluster represents a suspension over time (Day 0, 15, 30 and 60, when measurable). The size is presented on the positive y-axis, where the bars refer to the main peak (primary y-axis), together with the associated SD (half width of the peaks) and the mean of the single replicates (small dots, *n* = 2). The diamonds correspond to the PdI (secondary y-axis) mean, with associated SD. The ζ-potential stability is displayed on the negative y-axis, with corresponding SD and single replicates. Isotonic phosphate buffer was used in pre-dilutions for size measurements, while distilled water was used for the ζ-potential determination. Abbreviations: Lip refers to empty liposomes (gray); T (yellow) and C (blue) refer to the lipid dye and the surface lipid dye, respectively; PdI is the polydispersity index; SD is the standard deviation.

The next step was to determine the vesicle stability to assure that the experiments, performed with either fresh or week-old labeled liposomes, would not differ due to the freshness of their preparation. As evident in Figure 2A, no significant variations in size were detected (over 60 days) in liposomal suspension stored at 4 °C. Despite the initially neutral ζ-potential, the rather small size and narrow polydispersity of the liposomal suspension assured the stable size distribution over the tested timeframe. However, a progressive lowering of the ζ-potential over time was identified for all formulations, more evidently for the C-containing liposomes, suggesting that this increased surface charge could help stabilize the suspension at the later time points [12]. As expected, the long-term storage at 25 °C (Figure 2B) resulted in great instability of all dye-containing formulations as compared to their respective no-dye control. We were able to follow the size distributions for formulations stored at 25 °C for only 15 days; when measuring the formulations stored for 30 days, a dense sediment was observed in the vials and the suspension was not stable enough throughout the duration of the measurements. We could only measure the dye-free liposomes which were stable in size. Although the stability issue for storage at room temperature was expected, it also indicated that leaving the samples at room temperature could affect the vesicle aggregation and consequently, the cellular uptake, and it is one of the parameters which needs to be considered in the experiments.

**Figure 3:**
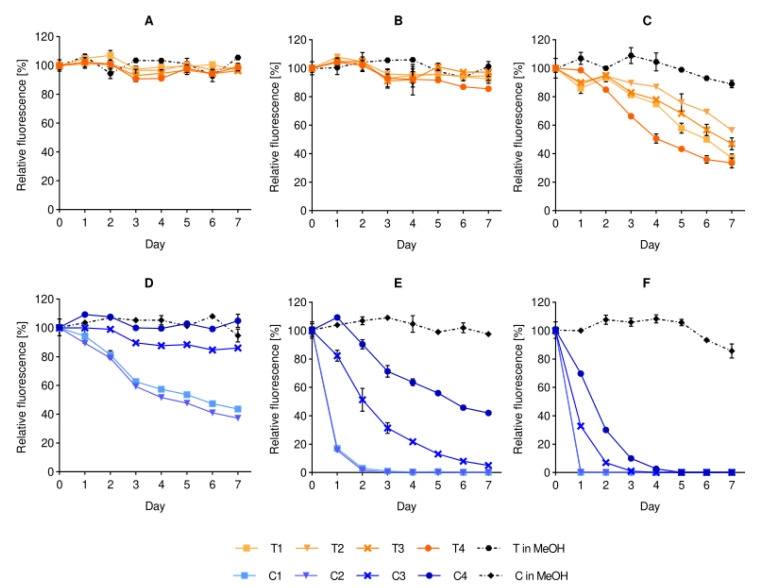
Effect of storage temperature on the fluorescence stability of liposomal dyes. The stability of T-containing formulations (panels **A**–**C**) and C-containing formulations (panels **D**–**F**) are compared to their respective dye solutions in methanol after daily sampling at the temperatures of 4 °C (**A**,**D**), 25 °C (**B**,**E**) and 37 °C (**C**,**F**). All formulations were pre-diluted in isotonic buffer to fit in the detectable range of the instrument. The fluorescence is expressed as mean (%) ± SD (*n* = 3), where 100% is the initial fluorescence value of each formulation (Day 0). Abbreviations: T refers to the lipid dye; C to the surface lipid dye; MeOH is short for methanol; SD is standard deviation.

After identifying the stability issue with dye-containing liposomes, we went a step further to evaluate the effect of storage temperature on the stability of the dye-containing formulations, focusing on the stability of fluorescence, namely the loss of fluorescence over time. As shown in Figure 3, the overall fluorescence stability of all T-containing formulations in buffer was higher than the corresponding C-containing liposomes. The trend was observed at all tested storage temperatures (4, 25 and 37 °C). Nevertheless, when the same dyes were dissolved in methanol and their stability was tested under the same storage conditions, the dyes remained stable. The medium of the liposomal suspension, thus, directly affected the fluorescence stability of the fluorophores in the formulations, and the degree of instability was dependent on the fluorophore and its localization in the bilayer. In our case, the greater instability of the C-lip formulations supports the expected localization of the fluorescent moiety onto the surface, as the carbocyanine group is known to be prone to photobleaching in buffer [13] when not protected by the lipid bilayer [14]. This finding has a significant impact and should raise concerns when explaining the biological fate of liposomes based on the measured intensities of liposome-associated dyes.

### 2.2. Biological Activity In Vitro

Another important parameter to be considered when choosing the dye to be incorporated in liposomes is the potential toxicity and pharmacological response of dye-in-liposomes. We tested the cytotoxicity and anti-inflammatory responses of all liposomal suspensions.

#### 2.2.1. Cytotoxicity

**Figure 4:**
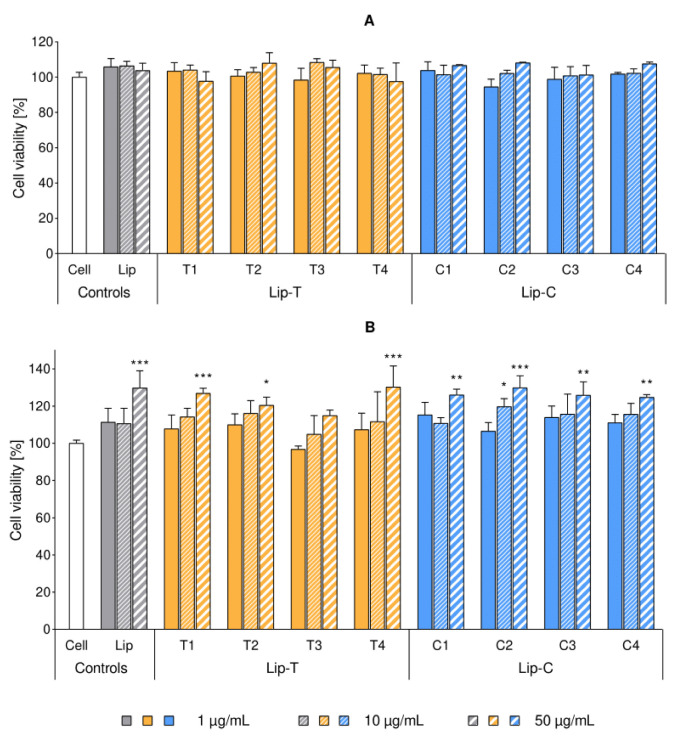
Cytotoxicity of liposomal suspensions tested on RAW 264.7 macrophages (panel **A**) and HaCaT keratinocytes (panel **B**). Cell proliferation was expressed in percentage, referencing the untreated control (mean ± SD, *n* = 3). Each cluster refers to the same liposome formulation, applied in three different concentrations indicated in the legend. Concentration values correspond to the concentration of lipids per well (1, 10 and 50 µg/mL, complete RPMI-1640). Stars of significance indicate the increased proliferation over cell control (white bar): * *p* < 0.05; ** *p* < 0.01; *** *p* < 0.001. All dye-containing liposomes affected the cell proliferation in a similar manner as empty liposomes. Abbreviations: Lip refers to liposomes; T and C refer to the lipid dye and the surface lipid dye, respectively; Lip-T and Lip-C denote the dye-containing formulation; SD is standard deviation.

Cytotoxicity of labeled liposomes were studied on macrophages (RAW 264.7) and keratinocytes (HaCaT) at lipid concentrations of 1, 10 and 50 µg/mL. None of the formulations exhibited any cytotoxic effect on the selected cell lines up to the lipid concentration of 50 µg/mL. Moreover, the behavior of the dye-containing liposomes did not significantly differ from the control represented by dye-free liposomes (Figure 4). The colorimetric reduction in the tetrazolium salt WST-8 was used as an indicator of cell viability, since this reaction requires an active dehydrogenase catalysis to be completed [15]. Hence, cell proliferation was expressed as a function of this enzymatic activity, setting 100% as the viability of the untreated control. Overall cell growth after the treatment was similar to the untreated controls, however, a tendency of increased keratinocytes proliferation was noticed for cells treated with liposomes in increasing lipid concentrations (Figure 4B).

#### 2.2.2. Anti-Inflammatory Assay

**Figure 5:**
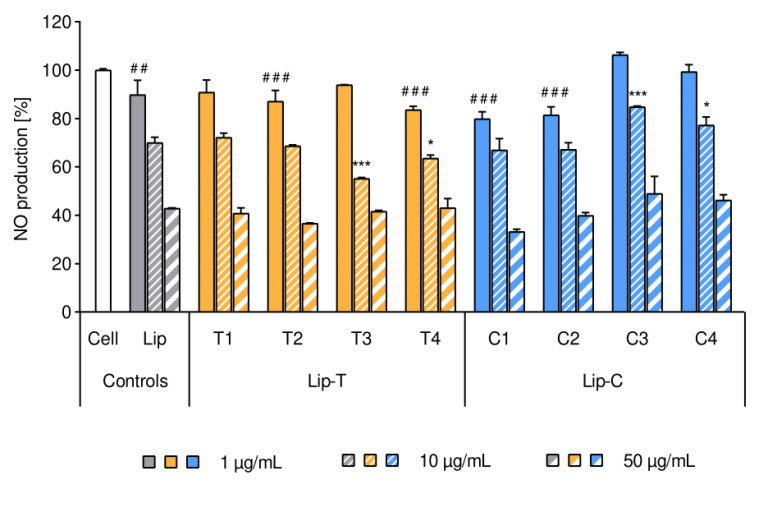
Effect of liposomes on the intrinsic inflammatory activity of RAW 264.7 macrophages. Intrinsic nitric oxide production was expressed in percentage from 0% (non-inflamed cells) to 100% (1 μg/mL LPS-inflamed cell as control, white bar). Bars represent mean ± SD (*n* = 3) and separate clusters refer to different liposomes in three concentrations of lipids (1, 10 and 50 µg/mL per well). Tags of significance show deviance from the cell control (white bar) for all the lowest lipid concentration applied (1 µg/mL): #: *p* < 0.05; ###: *p* < 0.001. All liposomes at the lipid concentration of 10 and 50 µg/mL showed the highest significance of *p* < 0.001 (not marked in the figure). Stars of significance indicate deviance from the respective empty liposome control (Lip, gray cluster): *: *p* < 0.05; ***: *p* < 0.001. Abbreviations: Lip refers to liposomes; Lip-T and Lip-C refer to the dye-containing liposomes with T (lipid dye) and C (surface lipid dye) respectively; NO is nitric oxide; SD is standard deviation.

Anti-inflammatory responses related to liposomal formulations were expressed by measuring the inhibition on nitric oxide (NO) production in LPS-induced macrophages. The anti-inflammatory activity was expressed as percentage of NO production inhibition calculated with respect to control (untreated cells as shown in Figure 5, white bar).

All liposomal formulations showed lipid concentration-dependent inhibition of NO production in LPS-activated macrophage (Figure 5) and the results were in agreement with the literature [16,17]. Liposomal formulations at the concentration of 50 µg/mL inhibited approximately 50% of NO production as compared to the non-treated control.

After the cells were exposed to liposomal suspensions, an evident reduction in the inflammatory activity was measured and validated by a significant concentration-dependency (*p* < 0.01). As Figure 5 shows, within each cluster, the increasing lipid concentration resulted in a decreased NO production. The pattern for the anti-inflammatory effect of non-labeled liposomes was similar to the fluorescently-labeled liposomes (Figure 5). Slight deviations (mostly not on significant level) were observed for both dye-containing liposomes when liposomes comprised higher dye to lipid ratios (T3, T4 and C3, C4, respectively).

#### 2.2.3. Flow Cytometry and Imaging

**Figure 6:**
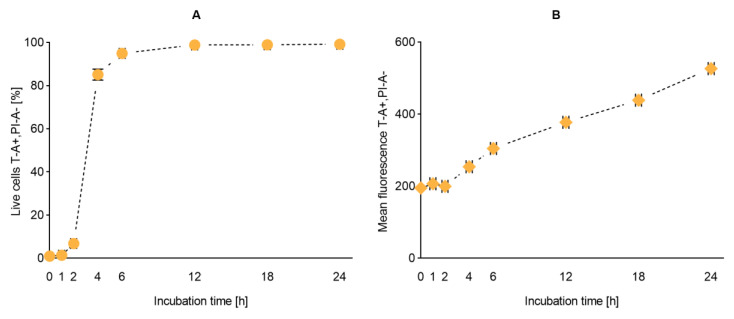
Uptake of T1 liposomes by RAW 264.7 macrophages. Liposomal uptake was expressed as percentage of fluoresce-activated live cells (panel **A**) and mean fluorescence (panel **B**). Each point represents mean ± SD on the y-axis (*n* = 3). A standard error of ± 5 min was given as default on the incubation time for the practical handling of the samples. Abbreviations: T refers to the lipid dye; T-A+, PI-A- represent the gate for fluorescence-activated live cells; SD is standard deviation.

After confirming the safety and retained biological activity of the liposomal suspensions, we proceeded to follow their cellular uptake. Based on the stability data, we have selected the T1 liposomes as model dye-in-liposomes suspension. T1 liposomes comprise the lowest dye-to-lipid ratio, which would be highly advantageous considering both cost and possible interference. Liposomal uptake was quantified and imaged by flow cytometry at different time points over 24 h. According to the number of cells screened as T-positive (T-A +), the uptake was proven in almost all cells within the first 6 h of incubation (Figure 6A). On the other hand, the total uptake—quantified as mean peak of fluorescence—did not reach a plateau in the 24 h of analysis, thus, indicating that no saturation took place in the tested timeframe (Figure 6B).

**Figure 7:**
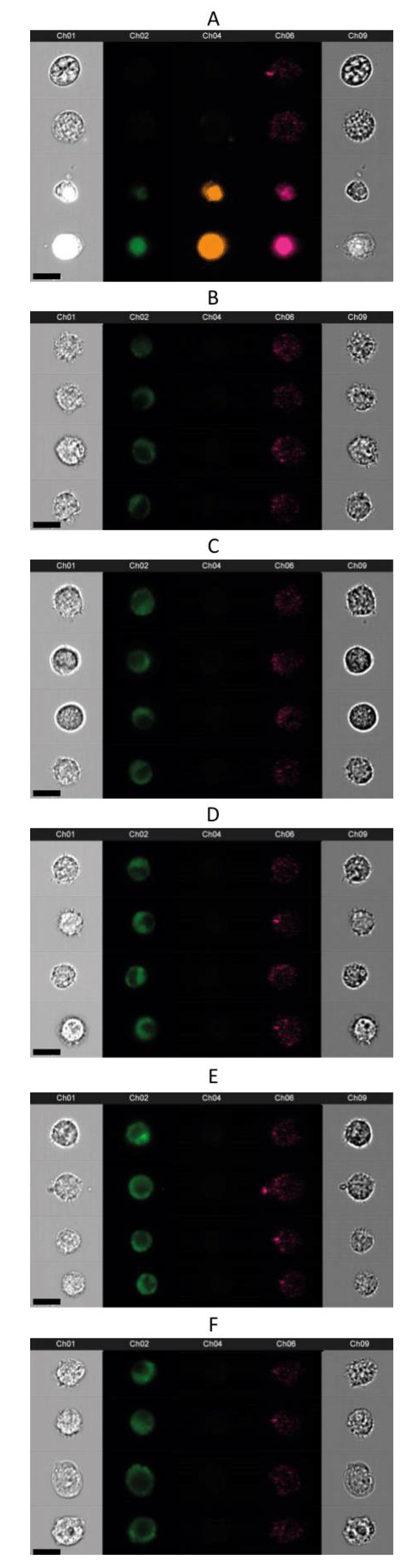
Uptake of T1 liposomes by RAW 264.7 macrophages presented by flow imaging. Four representative pictures are selected for the incubation time of 4 (panel **B**), 6 (**C**), 12 (**D**), 18 (**E**) and 24 h (**F**) after adjusting auto-fluorescence on the negative control (**A**: two upper rows). Ch01 and Ch09 exhibit bright field images, Ch02 detects the fluorophore T, Ch4 represents the PI fluorescence and Ch6 visually expresses the side scattering. A ch04-positive control is included for comparison (**A**: two bottom rows). The scale bar (black bar, bottom right) is 15 µm. Abbreviations: Ch refers to channel; PI to Propidium Iodide; T to the lipid dye.

The flow imaging analysis confirmed the cellular uptake, indicating a non-homogeneous distribution of the fluorescent signal within the cell. As expected, the highly lipophilic dye neither was able to reach the nucleus nor did it dissolve uniformly in the cytoplasm (Figure 7).

## 3. Discussion

Nanomedicine was expected to offer means to revolutionize diagnostics and targeted drug therapies, while improving the cost-effectiveness of health care. Although many of the promises remain to be fulfilled, success stories, even limited in numbers, confirmed its potential. Therefore, focus on addressing the challenges and current limitations might lead to a faster translation from laboratory into clinic [2,18]. There are various means to approach the challenges, from in vitro conditions to more complex in vivo studies. Recently, the intracellular delivery of nanomaterials as well as nanomaterial-associated drugs have attracted increasing attention due to the ever growing interest in subcellular drug targeting [19]. To be able to confirm the intracellular fate of nanoparticles, and optimize their properties to achieve subcellular delivery, we aimed to focus on a rather simple yet often neglected interplay between the nanocarrier and the dye used to follow its fate. To do so, we selected a simple liposomal composition based on the neutral (zwitterionic) phospholipid phosphatidylcholine. Vesicular size, surface charge and modifications, as well as lipid composition, are widely known to affect the liposome internalization rate and mechanism, with a consequent effect on intracellular dispatch [20,21,22,23,24]. However, it is the interplay of these features that determines the biological outcome. Thus, this work intended to provide deeper insight on the effects a fluorophore associated with liposomes can have on the dye-in-liposome system.

To avoid the known effect of size on the internalization behavior [25], all liposomal suspensions were thereby prepared to be of similar size, namely below 200 nm (Table 1). Plain (dye-free) liposomes remained stable in size over a period of 60 days when stored at 4 °C (Figure 2). The zeta-potential, originally slightly negative or neutral for both plain and dye-in-liposomes, indicated that neither of the dyes was interfering with the surface charge of the liposome (Table 1). However, the surface charge changed, exhibiting an increased negativity as observed over time at 4 °C storage, and potentially prolonging the stability of the suspension.

The findings were in agreement with previously reported low degree of lipid oxidation. Plain conventional liposomes are known to exhibit a slight oxidation tendency over time [26], which has been correlated to a decrease in zeta-potential [27]. Oxidation is indeed known to cause rearrangements of atoms and ions with steric changes of the orientation of lipid polar heads; the computational analysis even proposed the possibility of complete chain reversal of the oxidized phospholipids [28]. Since the zeta-potential measures the interaction of the liposomal surface with counter-ions in the medium, changes in the orientation of the choline zwitterion would result in different ζ-average values [29,30].

In this dynamic model, the presence of a fluorophore in the bilayer thus affects the behavior of the system according to its chemistry and location (Figure 1). The lipid dye (T) exhibited a low superficial interference and better stability against ionic changes in the environment, since this fluorescent moiety is known to reside stably within the core of the lipid bilayer [10]. The surface lipid dye (C), although not charged per se, exhibited a more relevant deviation from the control. This may be explained by the presence of an aromatic group with delocalized charge for interaction with the medium [14].

Interestingly, the characterization of all labeled formulations stored at 25 °C indicated a denser sedimentation over time in comparison to the plain liposomes control. This temperature-dependent physicochemical instability corresponded to the fluorescence instability of the dye in the formulations. The storage at higher temperatures (25 and 37 °C) yielded a progressive decrease in fluorescent signal for all of the dye-containing liposomes. This decrease was not observed to the same extent in the controls, namely the dyes dissolved in methanol, suggesting a direct effect of the medium on the fluorescence stability of the formulation. In the case of the carbocyanine derivative (C), the lower stability in the intact formulation is explained by its known quenching and lower fluorescence in phosphate-buffered saline PBS [13]. When comparing the observed behavior with previously published works on correspondent hydrophilic cyanines, our results are similar to the findings on cyanines freely dissolved in PBS, compared to the cyanines inside the bilayer [14,31]. Although we do not have precise evidence, the findings further corroborate the postulation on the surface localization of the fluorescent moiety in our C dye (Figure 1D). On the contrary, the lipid dye (T) showed a limited reduction in fluorescent signal over time and with increased temperature, which suggested an overall better fluorescence stability, in agreement with published research [11,32]. Remarkably, for both dyes, the fluorophore concentration did affect the (in)stability of the formulation. It was expected that the higher the dye-to-lipid ratio was, the greater the chance to retain a relevant fluorescent signal after storage/incubation would be. This was not verified for the lipid dye (T) as the highest ratio dye-to-lipid exhibited a higher loss of fluorescence signal over time. Hence, the bilayer exerts a physicochemical protection on the fluorophore from the environment (here, in T1, T2 and T3) but a spatial saturation (here, reached in T4) can reduce this protective effect, increasing the exposure of the fluorescent moieties [14,28].

The small radical NO was used as an indicator of the inflammatory response in macrophages as it is a potent inflammatory mediator [33]. Specifically, the LPS used in the present study activated the conversion of L-arginine into L-citrulline with the production of NO as a major byproduct. This unstable radical was quickly converted into NO_3_^−^ and NO_2_^−^, molecule that the Greiss reagent can quantify through a colorimetric reaction. Hence, the quantification of the colored product was first correlated to the NO initial concentration through a NaNO_2_ standard curve, and then, correlated to the inflammatory activity of the macrophages [34].

The presence of dye within liposomes did not affect their cytotoxicity, as indicated in Figure 4. Moreover, no difference in cell proliferation was detected when the cells were treated either with dye-containing or dye-free liposomes. Our findings were in full agreement with earlier studies involving keratinocytes with a concentration-dependent increase in cellular proliferation [15]. Keratinocytes are responsible for the maintenance of the skin barrier where extracellular lipids play a major role [35] and are, therefore, a good model to test potential toxicity. As a second type of cells, we selected macrophages due to their role in interacting with nanocarriers and wide variety of inflammatory diseases [36]. We could not detect liposome-induced proliferative effects in non-inflamed cells (Figure 4A), however, we confirmed a concentration-dependent reduction in NO production after liposomal treatment of LPS-inflamed cells (Figure 5) [16,17]. Although no toxicity was determined for the dye-containing liposomes, we observed larger variability in the readings for NO production in activated macrophages. This was more pronounced for the surface lipid dye (C) and at the highest labeling ratios.

T1 was selected as most suitable formulation for a time-course internalization assay. Our rationale was that if we could successfully follow the fate of liposomes with the least dye content, we would limit possible interference of the dye with biological processes. A weaker fluorescent signal was already registered in the first two hours of incubation, as expected for neutral plain liposomes [37], but almost all cells were screened as positive to the fluorophore after 6 h. In spite of the sharp rising in the number of cells that had internalized liposomes within 2 and 6 h, the progressive linear increase in mean fluorescence confirmed that the internalization capacity of the macrophages was not saturated. These internalization profiles were confirmed in flow imaging and, as expected, the fluorophore did not uniformly distribute in the cytosol and was condensed to intracellular organelles, such as phagosomes and phagolysosomes. Phagocytosis has long been considered the main internalization pathway for liposomes encountering macrophages [20]. Moreover, the presence of a protein phospholipid receptor on the lysosomal membrane is known to facilitate the liposomal recognition by these organelles [38].

When characterizing a labeled nanoscale system such as dye-in-liposomes, the interplay of the different components and the environment can affect not only the leakage of the dye, but also the final biological activities of the system. Therefore, fluorophores that have shown lower tendency to detach from their system (e.g., Cy5-DSPE, [8]) could have unwanted biological effects and/or higher instability of the fluorescent signal, which could explain lack of internalization [6] as possible false-negative results. On the contrary, higher physicochemical stability of the components in the nanosystem (e.g., TopFluor^®^-PC [32]) can then show higher leakage of the fluorophore, with a consequent loss of tracking confidence over time [8].

To summarize, a deep characterization of the nanosystems can be performed by addressing the interplay between its different components. In fact, not only the presence of the dye can affect the physicochemical properties of the system (such as size and zeta potential), the different components within the system can also affect the properties of the dye. Additionally, the fluorescent signal and behavior of the fluorophore in a system can affect both its physicochemical, as well as its biological properties, modulated by a consequent variation of the experimental conditions.

A trade-off has to be considered when choosing a fluorescent dye for a formulation. As a general consideration, time and temperature are the first in line to affect the system stability. Only then, once the effect of different sizes and zeta-potentials are removed, can the steric organization on the nanoscale and the compatibility of the fluorophores with the medium become the predictors of the usability of the system. Leakage information will then be fundamental when designing experiments over selected time frames. This full assessment is necessary for the validation of fluorescence-based assays in the screening of targeting efficiency not only to avoid false-positive results but also not to disregard false-negative promising strategies.

## 4. Materials and Methods

### 4.1. Materials

Soy phosphatidylcholine (Lipoid S100, SPC) was generously provided by Lipoid GmbH (Ludwigshafen, Germany). The 1-palmitoyl-2-(dipyrrometheneboron difluoride)undecanoyl-sn-glycero-3-phosphocholine (TopFluor^®^-PC, T) and 1,2-distearoyl-sn-glycero-3-phosphoethanolamine-N-(Cyanine 5) (18:0 Cy5-PE, C) were purchased from Avanti Polar Lipids, AL, USA. Methanol, naphthylethylenediamine, potassium phosphate monobasic, sodium chloride, sodium phosphate dibasic dodecahydrate, sulfanilamide, phosphoric acid (H_3_PO_4_), RPMI-1640 medium, Dulbecco’s phosphate buffer, lipopolysaccharide (LPS, Escherichia coli, 055:B5) and propidium iodide (PI) were obtained from Sigma-Aldrich, Steinheim, Germany.

### 4.2. Liposome Preparation

The film hydration method was used to prepare liposomes in aqueous medium [39]. Briefly, lipids were dissolved in methanol in a round-bottomed flask. To form a thin lipid film, low-pressure rotary evaporation was performed on a Büchi Rotavapor R-124 with vacuum pump V-700 (Büchi Labortechnik, Flawil, Switzerland). The film was then resuspended in 8 mL of isotonic phosphate buffer (pH 7.4, 290 mOsm) by hand-shaking and 10 min bath sonication (Bransonic^®^ ultrasonic 5510, Vlierberg, Holland). A concentration of 10 mg/mL of neutral SPC S100 (with over 94% of pure phosphatidylcholine from soybean) was used for all formulations. Fluorescent dyes (T and C) were separately incorporated in the initial lipid mixture at the concentrations of 0.015, 0.030, 0.060 and 0.120 mg/mL, respectively.

The effective size reduction of the multilamellar dispersions was achieved by combining 2 min sonication (Ultrasonic processor 500 W, Sigma-Aldrich, MO, USA) and a stepwise hand extrusion through Nucleopore^®^ polycarbonate membranes (with sieving sizes of 400 and 200 nm, respectively). Overnight stabilization was allowed in between the steps and prior to the characterization. All liposomal suspension were then stored in the fridge (4 °C) and at room temperature (25 °C), respectively.

### 4.3. Liposome Characterization

#### 4.3.1. Size Analysis

Photon correlation spectroscopy was used for the size distribution analysis of liposomes in suspension, as previously described [40]. Submicron particle sizer model 370 (Nicomp Santa Barbara, CA, USA) was set on the vesicle mode and intensity-weighed distribution. All suspensions were diluted in isotonic phosphate buffer to obtain a particle intensity of 400–500 kHz for 15 min/cycle. The sample measurement was repeated on day 0, 15, 30 and 60 to follow the size stability dependency on the storage temperature (4 or 25 °C).

#### 4.3.2. Ζeta-Potential Analysis

The zeta-potential distribution was derived from a Gaussian distribution analysis of the electrophoretic mobility, as an indication of liposomal surface charge [39]. Laser Doppler Electrophoresis was applied utilizing a Malvern Zetasizer Nano—ZS (Malvern, Oxford, UK) in the General Purpose mode. All suspensions were diluted 1:20 in deionized water and measured after 15 min equilibration time. Deionized water was chosen over buffer and tap water to ensure the reliability of the measurements, as the presence of the fluorophore tends to interfere with the instrument sensitivity [41]. To further stabilize the measurements, individual runs were performed with a 1 min pause in between to allow the electrodes not to be overheated for high mobility samples. Stability measurements were conducted on day 0, 15, 30 and 60, respectively.

### 4.4. Fluorescence Analysis

Fluorescence stability of all dye-containing formulations was assessed as a function of storage temperature. All suspension were diluted 1:100 in isotonic phosphate buffer and stored separately in the fridge (4 °C), at room temperature (RT, 25 °C) and in the incubator (Termaks, Bergen, Norway) at 37 °C. Sampling for top fluorescence intensity reading was performed with a recurrence of 24 h for 7 days (Tecan SPARK spectrofluorometer, Tecan, Switzerland). Gain and wavelength (λ) of excitation (ex) and emission (em) were optimized on the full scan of the formulations, on Day 0, and maintained throughout the experiment (T: λex = 430 nm, λem = 510 nm, C: λex = 600 nm, λem = 665 nm; gain = 90). The same procedure was used to read each dye’s own stability in methanol.

### 4.5. Biological Activity In Vitro

Murine macrophages RAW 264.7 (ATCC^®^ TIB-71TM, ATCC, Manassas, USA) and human immortalized keratinocytes HaCaT (ATCC, Manassas, USA) were cultured in flasks and incubated at 37 °C with 5% CO_2_. RPMI-1640 medium was used to culture the cells, supplemented with the addition of 10% (v/v) fetal bovine serum and antibiotics (penicillin-streptomycin). Complete medium was also used to pre-dilute all liposomal formulations right before treating the cells [15,39].

#### 4.5.1. Cytotoxicity Assay

Cell toxicity of all formulations was evaluated using the Cell Counting Kit-8 (Sigma-Aldrich Chemie) as a function of dehydrogenase activity, directly proportional to cell viability in macrophages (RAW 264.7) and keratinocytes (HaCaT) [15]. To 90 µL/well of cell suspension (1 × 10^5^ cells/mL, pre-incubated overnight in 96 wells), 10 µL of either medium (control) or liposomes were added. Three concentrations of liposomes (1, 10 and 50 μg/mL of lipids), each in triplicates, were exposed to the cells to observe the cytotoxic effect. At the 24 h endpoint, CCK-8 reagent (10 µL/well) was applied and plates were further incubated for 4 h. The detection of the absorbance was set on 450 nm and referenced at 650 nm (Tecan SPARK spectrophotometer, Tecan, Switzerland).

#### 4.5.2. Anti-Inflammatory Activity

The anti-inflammatory effects of all liposomal formulations were analyzed on LPS-activated macrophages (RAW 264.7) by measuring the inhibition on the nitric oxide production [39]. Cells were cultured in RPMI-1640 supplemented medium until the formation of a confluent monolayer. The homogenous cell suspension (5 × 10^5^ cells/mL) was plated into a 24-well plate (1 mL/well) and incubated for 24 h. The old medium was replaced with 990 µL of LPS-containing medium (1 µg/mL of LPS) in order to induce inflammation. All formulations (10 µL each) were applied in triplicates for three different concentration of lipids (1, 10 and 50 μg/mL per well, respectively). After 24 h incubation, the anti-inflammatory response was quantified by measuring NO production in the medium by the cells with the Greiss reagent (1% sulfanilamide, 0.1% naphthylethylenediamine, 2.5% H_3_PO_4_). Spectrophotometric endpoint measurements were performed at 540 nm and evaluated through a NaNO_2_ standard curve (Single Cuvette UV Vis Spectrophotometer, SpectraMax 190 with SoftMax Pro v5 software, Molecular devices, CA, USA).

#### 4.5.3. Flow Cytometry

The time dependency of liposomal uptake in macrophages was quantified by flow cytometry (FACSAriaTM with FACSDiva software version 8.0.1, BD Biosciences, San Jose, CA, USA) Macrophages (RAW 264.7) were seeded on 6-well plates 24 h prior to the treatment (3 mL/well). Liposomes (T1) were freshly pre-diluted in complete RPMI-1640 (50 µg/mL per well) and the suspension was used to change the medium in each well. At the endpoints of 1, 2, 4, 6, 12, 18 and 24 h, cells were washed with Dulbecco’s phosphate buffer and suspended in 800 µL of fresh RPMI-1640 by pipetting. Propidium iodide (PI) was added to the filtered cell suspensions to single out live cells, which were gated as PI-negative (PI-A-). Ten thousand events were recorded and liposomal uptake was measured on the correspondent channel (T-A) after adjusting for cell/medium auto-fluorescence of the untreated control [42].

#### 4.5.4. Flow Imaging

Fluorescence-activated cell sorting was performed on the T-A+, PI-A- population, which was concentrated and visualized with a flow imaging system (ImageStreamX^®^ with IDEAS software version 6, Amnis, Seattle, WA, USA). Cells were gated in the forward vs. side scattering diagram to exclude the signal from running calibration beads. Live cells were then located as low Channel 4 intensity (Ch4, 610/30 nm) and 100 pictures were recorded with 60X magnification and max sensitivity. Both cameras were activated to obtain bright field images and confirm the presence of a cell in the stream (Ch1 and Ch9). Furthermore, side scattering was recorded to confirm the granularity of macrophages (Ch6, 762/35). The liposome uptake was imaged in green fluorescence (Ch02, 528/65 nm) after 4, 6, 12, 18 and 24 h. Untreated live and dead cells were also imaged, as negative and positive controls, respectively, to adjust for auto-fluorescence and validate the gating tree [43].

### 4.6. Statistical Analysis

One-way ANOVA with Bonferroni’s multiple comparisons post test was used to assess statistical significance. Significance was assigned for *p* value < 0.05 (GraphPad Prism version 8.1.2 for Windows, GraphPad Software, La Jolla CA, USA).

## 5. Conclusions

A thorough physicochemical and biological characterization of dye-containing nanosystems is required to fully benefit from the enormous potential of the fluorescence-based techniques in tracing the fate of nanocarriers. When introducing a fluorescent dye, the individual properties of both free dye and unlabeled nanocarrier, as well as their stability and behavior, can be affected by the experimental conditions, namely medium, temperature, time, dye localization and concentration. Therefore, deeper characterization of the dye-containing nanocarriers can assure the interpretation accuracy of the fluorescence-based assays. This can be considered the key to success in optimizing new drug delivery systems.

## Figures and Tables

**Table 1:** Characteristics of freshly prepared liposomal suspensions.

Type of Liposomes	Fluorescent Dye		Vesicle Size			ζ-Potential
	T [mg/mL]	C [mg/mL]	Peak 1 [nm (%)]	Peak 2 [nm (%)]	PdI	[mV]
Lip	-	-	135 ± 1 (88%)	38 ± 1 (12%)	0.23 ± 0.01	-1.4 ± 0.1
T1	0.015	-	167 ± 2 (95%)	39 ± 11 (5%)	0.17 ± 0.01	−2.0 ± 0.2
T2	0.030	-	158 ± 7 (96%)	35 ± 12 (4%)	0.16 ± 0.01	−3.6 ± 0.3
T3	0.060	-	160 ± 4 (95%)	35 ± 4 (5%)	0.20 ± 0.04	−1.2 ± 0.1
T4	0.120	-	168 ± 4 (89%)	42 ± 3 (11%)	0.22 ± 0.01	−1.8 ± 0.0
C1	-	0.015	163 ± 8 (94%)	35 ± 1 (6%)	0.21 ± 0.01	−1.2 ± 0.1
C2	-	0.030	161 ± 4 (89%)	40 ± 1 (11%)	0.24 ± 0.01	−1.2 ± 0.1
C3	-	0.060	156 ± 1 (93%)	37 ± 1 (7%)	0.22 ± 0.01	−2.0 ± 0.1
C4	-	0.120	157 ± 7 (89%)	39 ± 8 (11%)	0.22 ± 0.01	−1.5 ± 0.1

Values are expressed as mean ± SEM (*n* = 2). To describe the bimodal size distribution, the intensity-weighted percentage of each population peak is indicated in brackets. The vesicle size was measured after dilution in isotonic buffer, whereas ζ-potential after dilution in distilled water. The concentration of neutral (zwitterionic) SPC (>94% purity) was 10 mg/mL. Abbreviations: SPC refers to soy phosphatidylcholine; T represents TopFluor lipid dye whereas C represents Cy5-DSPE surface lipid dye; PdI is polydispersity index; SEM, standard error of the mean.

## Abbreviations

C	1,2-distearoyl-sn-glycero-3-phosphoethanolamine-N-(Cyanine 5) (18:0 Cy5-PE), surface lipid dye
CCK-8	Cell Counting Kit-8 (Sigma-Aldrich Chemie)
MW	Molecular weight
NO	Nitric oxide
PdI	Polydispersity index
PI	Propidium iodide
RT	Room temperature, 25 °C
SPC	Soy phosphatidylcholine (Lipoid S100)
T	1-palmitoyl-2-(dipyrrometheneborondifluoride)undecanoyl-sn-glycero-3-phosphocholine (TopFluor^®^-PC), lipid dye
WST-8	Water-soluble tetrazolium salt, main reagents of the Cell Counting Kit-8 (Sigma-Aldrich Chemie, Patent No. WO97/38985); [2-(2-methoxy-4-nitrophenyl)-3-(4-nitrophenyl)-5-(2,4-disulfophenyl)-2H-tetrazolium,monosodium salt]
